# Point-of-care testing in companion and food animal disease diagnostics

**DOI:** 10.3389/fvets.2022.1056440

**Published:** 2022-11-25

**Authors:** Binu T. Velayudhan, Hemant K. Naikare

**Affiliations:** ^1^Athens Veterinary Diagnostic Laboratory, College of Veterinary Medicine, University of Georgia, Athens, GA, United States; ^2^Tifton Veterinary Diagnostic and Investigational Laboratory, College of Veterinary Medicine, University of Georgia, Tifton, GA, United States

**Keywords:** point-of-care, veterinary, animal, disease, diagnostics

## Abstract

Laboratory diagnoses of animal diseases has advanced tremendously in recent decades with the advent of cutting-edge technologies such as real-time polymerase chain reaction, next generation sequencing (NGS), matrix-assisted laser desorption/ionization time—of—flight mass spectrometry (MALDI-TOF MS) and others However, most of these technologies need sophisticated equipment, laboratory space and highly skilled workforce. Therefore, there is an increasing market demand for point-of-care testing (POCT) in animal health and disease diagnostics. A wide variety of assays based on antibodies, antigens, nucleic acid, and nanopore sequencing are currently available. Each one of these tests have their own advantages and disadvantages. However, a number of research and developmental activities are underway in both academia and industry to improve the existing tests and develop newer and better tests in terms of sensitivity, specificity, turnaround time and affordability. In both companion and food animal disease diagnostics, POCT has an increasing role to play, especially in resource-limited settings. It plays a critical role in improving animal health and wellbeing in rural communities in low- and middle-income countries. At the same time, ensuring high standard of quality through proper validation, quality assurance and regulation of these assays are very important for accurate diagnosis, surveillance, control and management of animal diseases. This review addresses the different types of POCTs currently available for companion and food animal disease diagnostics, tests in the pipeline and their advantages and disadvantages.

## Introduction

Point-of-care testing (POCT) is defined by International Standard ISO 22870 as testing that is performed near or at the site of a patient with the result leading to possible change in the care of the patient. The test is performed outside of a diagnostic laboratory near the patient with a rapid turnaround time. This enables rapid decision making and faster care or treatment of the patient. The POCT has been called by many names in human testing such as patient focused testing, near patient testing, near or next to the patient testing, and bedside testing in humans. In animal diagnostics, the POCT has been called as pen-side, animal-side, farm-side, barn-side or flock-side testing.

Literature on POCT began in 1984 with self-monitored blood glucose tests to monitor diabetes in people. As of August 1, 2022, there are 23,802 hits when you search for “point-of-care test” on Google search engine. This review is focused on POCT for animal infectious diseases. The keywords used for literature search were point-of-care test/testing, POCT, animal disease, veterinary, infection/infectious, pathogen, and diagnosis.

A point-of-care testing can be done in a number of places or areas. In human medicine, this could be the patient's home, clinic or health care provider's office, emergency care, or in a public place such as an airport or conference center. In the case of animal health, this could be the farm or flock side or pet's home, a veterinary hospital or a veterinarian's office or a common place. On the human side, there are a number of POCT currently available to test for electrolytes, blood gases, cholesterol, cardiac enzymes such as troponin, abuse of drugs such as barbiturates or benzodiazepines, and various infectious agents or their antibody response. Regulatory oversight and quality control (a well-organized quality assurance program) are critical components for the reliability and sustainability of POCTs whether these tests are used for surveillance, outbreak, regulatory or recovery testing (1).

There are a number of ways by which POCT can be classified such as based on analytes tested, technologies and methodologies used (2). In this review classification of tests based on three analytes, i.e., antigen, antibody and nucleic acid is discussed. Per a 2022 BioSpace Report (3), Veterinary POCT market was valued at around $2.15 billion in 2020. It is estimated to grow at a 12.3% rate from 2021 to 2030 and projected to be worth $5.69 billion by 2030 (3). This rapid growth is augmented by evolving technology and increasing market need to respond to new and emerging diseases including the ongoing COVID-19 pandemic. In veterinary diagnostics, the companion animal market is more receptive to new POCTs even if they are expensive since cost is not a primary deciding factor here compared to food animal industry (4).

## Antigen based assays

Antigen and antibody based POCT are immunoassays that are available for testing many animal diseases and for animal health monitoring. The most widely used immunoassay configuration is the enzyme-linked immunosorbent assay (ELISA) as they are easy to develop, easy to use and easy to validate compared to nucleic acid based assays (5). However, their specificity and sensitivity are often inferior to nucleic acid-based assays.

Lateral flow assays are commonly used in POCT as an easy-to-use method to read results by the end users, especially for those who are not technology-savvy. There are several advantages to lateral flow assays including relative easiness to manufacture, scalability, simple to use, easy to read the results, suitable to use in any environment and for any applications whether in veterinary diagnostics, food industry, or human health and disease monitoring.

The earliest antigen based POCT in animal disease diagnostic was the antigen detection assay developed for canine dirofilariasis in 1983–84 period (6–8). This paved the way for additional POCTs that utilized either antigen capture or antibody detection methods in companion animals (6). This was further accelerated by the introduction of lateral flow immune assays and SNAP (a registered trademark of IDEXX Laboratories, Inc.) technology (9, 10). The SNAP assay is an in-clinic device that performs each of the ELISA steps in a timed sequential manner with minimal consumer interface. An antigen SNAP assay for the detection of heartworm became available since 1990s (10). Currently, there are a number of SNAP tests available for the detection of antigens and antibodies from a wide variety of infectious diseases (10). Lateral flow immunoassays are currently available for canine parvovirus, *Ehrlichia, Giardia*, Lyme disease, feline leukemia, *Cryptococcus*, and feline immunodeficiency (4, 11–13). A parvovirus SNAP ELISA (IDEXX Laboratories Inc.) has been used as a reliable POCT to diagnose feline panleukopenia and the test has comparable specificity but less sensitivity compared to feline parvovirus PCR (14).

On the avian side several tests are available for influenza A virus in chickens, ducks, turkeys and geese. These include FluDirect Avian (Zoetis), BinaxNow influenza A+B (Alere), QuickVue influenza A+B and Sofia Influenza A+B (Quidel Corporation, CA), and Directigen EZ Flu A+B, and BD Veritor system (Becton, Dickinson, Sparks, MD). Most of these assays were originally developed for human testing but they have been used for avian applications as well. Antigen based POCTs have also been available for other avian pathogens including *Salmonella*, infectious bronchitis virus, infectious bursal disease and Newcastle disease virus (4, 15).

A wax-printed paper-based ELISA was developed by Zhao et al. (16) that utilized a microfluidic paper-based based analytical device for the detection of *Escherichia coli* O157:H7 from beef. The assay took <3 h to complete and used 5 μl of sample. The specificity, sensitivity, and repeatability of the assay was very similar to cELISA (16). Kelley et al. (17) evaluated the Alere Determine TB (tuberculosis) lipoarabinomannan antigen (LAM-test) and the Lionex Animal TB Rapid test using milk and urine samples from cattle and the results were mixed. The LAM-test, which is a WHO endorsed POCT in urine was not suited for testing samples from cattle whereas the Lionex assay yielded comparable results when bovine milk samples were tested (17). An antigen based POCT for the detection of Trypanosoma was developed using TcoB and TvGM6 antigens for *T. congolense* and *T. vivax*, respectively (18). The assays showed 92.0% sensitivity for *T. congolense* and 98.2% sensitivity for *T. vivax* while the specificity was 95.9% for both (18). A list of selected POCTs for diseases in companion animals is given in Table 1, and that of food animals is given in Table 2. A list of selected manufacturers of POCTs is given in Table 3.

**Table 1:** Commercially available POCT diagnostics for infectious diseases of companion animals^◇^.

**Species**	**Agent(s) Detected**	**Disease**	**Test Method**	**Manufacturer**
Canine Canine Canine Canine Canine Canine Canine Canine Canine Canine Canine Canine Canine Canine Canine Canine Canine Canine Canine Feline Feline Feline Feline Feline Feline Feline Feline Feline Feline Feline Feline Feline Feline Feline Feline Feline Equine Equine Equine Equine Equine Equine Equine Equine Equine	*Anaplasma phagocytophilum* and/or *A. platys* *Anaplasma platys* *Babesia gibsoni* *Bordetella bronchiseptica* *Borrelia burgdorferi* *Borrelia burgdorferi* Canine adenovirus 2 Canine distemper virus Canine distemper virus Canine giardia Canine herpes virus Canine influenza virus Canine parainfluenza virus Canine parvovirus Canine parvovirus Canine parvovirus Canine respiratory coronavirus *Ehrlichia canis, E. chaffeensis*, and *E. ewingii* *Ehrlichia canis* *Dirofilaria immitis* *Chlamydia felis* *Cytauxzoon felis* *Dirofilaria immitis* *Dirofilaria immitis* Feline calicivirus Feline coronavirus Feline coronavirus Feline herpesvirus Feline immunodeficiency virus Feline immunodeficiency virus Feline immunodeficiency virus Feline leukemia virus Feline leukemia virus Feline leukemiavirus Feline parvovirus *Mycoplasma felis* *Toxoplasma gondii* Babesia caballi Equine arteritis virus Equine herpesvirus 1 Equine herpesvirus 3 Equine herpesvirus 4 Equine influenza virus *Streptococcus equi* *Taylorella equigenitalis* *Theileria equi*	Anaplasmosis Anaplasmosis Babesiosis Kennel cough Lyme disease Lyme disease Kennel cough Canine distemper Canine distemper Giardiasis Canine herpes Canine influenza Canine parainfluenza Canine parvo Canine parvo Canine parvo Respiratory corona Ehrlichiosis Ehrlichiosis Canine heartworm disease Chlamydiosis Cytauxzoonosis Feline heartworm disease Feline heartworm disease Feline calici Feline corona/Feline infectious peritonitis Feline corona Feline herpes Feline immunodeficiency Feline immunodeficiency Feline immunodeficiency Feline leukemia Feline leukemia Feline leukemia Feline parvo Mycoplasmosis Toxoplasmosis Babesiosis Equine viral arteritis Equine rhinopneumonitis Coital exanthema Equine rhinopneumonitis Equine influenza Strangles Contagious equine metritis Equine piroplasmosis	Antibody LFA iiPCR iiPCR iiPCR Antibody LFA iiPCR iiPCR Antibody LFA iiPCR Antigen MICG~ iiPCR iiPCR iiPCR Antigen LFA Antigen MICG iiPCR iiPCR Antigen IFA iiPCR Antigen LFA iiPCR iiPCR Antigen LFA iiPCR iiPCR Antibody MICG iiPCR iiPCR Antibody MICG Antibody LFA iiPCR Antigen IFA Antigen MICG iiPCR iiPCR iiPCR iiPCR iiPCR iiPCR iiPCR iiPCR iiPCR iiPCR iiPCR iiPCR iiPCR	Zoetis GeneReach GeneReach GeneReach Zoetis GeneReach GeneReach Zoetis GeneReach Virbac BVT GeneReach GeneReach GeneReach Zoetis Virbac BVT GeneReach GeneReach Zoetis GeneReach Zoetis GeneReach GeneReach Zoetis GeneReach GeneReach Virbac BVT GeneReach GeneReach Virbac BVT Zoetis GeneReach Zoetis Virbac BVT GeneReach GeneReach GeneReach GeneReach GeneReach GeneReach GeneReach GeneReach GeneReach GeneReach GeneReach GeneReach GeneReach

◇ This is not a comprehensive list of all commercially available POCT assays. Please refer to the individual manufacturer's websites. Some of the POCT assays are available in only certain countries.

~MICG: Membrane immunochromatography.

**Table 2:** Commercially available POCT diagnostics for infectious diseases of food animals^◇^.

**Species**	**Agent(s) detected**	**Disease**	**Test method**	**Manufacturer**
Bovine	Bovine viral diarrhea virus	Bovine viral diarrhea	Antigen SNAP	IDEXX
Bovine	Bovine viral diarrhea virus 1&2	Bovine viral diarrhea	iiPCR	GeneReach
Bovine	Bovine leukemia virus	Bovine leukosis	iiPCR	GeneReach
Bovine	Bovine popular stomatitis virus	Bovine popular stomatitis	iiPCR	GeneReach
Bovine	*Mycobacterium bovis*	Bovine tuberculosis	iiPCR	GeneReach
Bovine	*Brucella abortus*	Brucellosis	iiPCR	GeneReach
Bovine	Bovine herpesvirus 1	Infectious bovine rhinotracheitis	iiPCR	GeneReach
Bovine	*Coxiella burnetti*	Q fever	iiPCR	GeneReach
Bovine	Rotavirus A	Calf Diarrhea	iiPCR	GeneReach
Bovine	*Tritrichomonas fetus*	Trichomoniasis	iiPCR	GeneReach
Poultry	Avian leukosis virus-J	Avian leukosis	iiPCR	GeneReach
Poultry	Avian influenza virus A	Avian flu	iiPCR	GeneReach
Poultry	Avian metapneumovirus	Avian rhinotracheitis	iiPCR	GeneReach
Poultry	Avian reovirus	Viral arthritis	iiPCR	GeneReach
Poultry	Chicken anemia virus	Infectious anemia	iiPCR	GeneReach
Poultry	Infectious bronchitis virus	Infectious bronchitis	iiPCR	GeneReach
Poultry	Infectious bursal disease virus	Infectious bursal disease	iiPCR	GeneReach
Poultry	Gallid alphaherpesvirus 1	Infectious laryngotracheitis	iiPCR	GeneReach
Poultry		Marek's disease		
Poultry	Gallid alphaherpesvirus 2	Newcastle disease	iiPCR	GeneReach
Swine	Newcastle disease virus	African swine fever	iiPCR	GeneReach
Swine	African swine fever virus	African swine fever	iiPCR	GeneReach
Swine	African swine fever virus	Swine dysentery	Immunochromatographic assay	Tetracore
Swine	*Brachyspira hyodysenteriae*	Hog cholera		
Swine	Classical swine fever virus	Foot and mouth disease	iiPCR	GeneReach
	Foot and mouth disease virus	Proliferative enteropathy	iiPCR	GeneReach
Swine	*Lawsonia intracellularis*	Porcine enzootic pneumonia	iiPCR	GeneReach
	*Mycoplasma hyopneumoniae*	Multisystemic diseases	iiPCR	GeneReach
Swine	Porcine circovirus 2	Porcine diarrhea	iiPCR	GeneReach
Swine	Porcine epidemic diarrhea virus		iiPCR	GeneReach
Swine	Porcine reproductive and respiratory syndrome virus	Porcine reproductive and respiratory syndrome (PRRS)	iiPCR	GeneReach
Swine	Pseudorabies virus	Pseudorabies	iiPCR	GeneReach
Swine	Senecavirus A	Seneca valley fever	iiPCR	GeneReach
Swine	*Streptococcus suis*	Multisystemic disease in pigs	iiPCR	GeneReach
Swine		Anthrax	iiPCR	GeneReach
Swine	*Bacillus anthracis*	Brucella		
Multiple species^#^	*Brucella* Spp.	Glanders	LFA (singleplex/multiplex)	Eurofins
	*Burkholderia mallei*	Melioidosis	LFA (singleplex/multiplex)	Eurofins
	*Burkholderia psuedomallei*		LFA (singleplex/multiplex)	Eurofins
	Coronavirus	Enteric and respiratory disease	LFA (singleplex/multiplex)	Eurofins
	Cryptosporidium parvum	Cryptosporidiosis	Antigen MICG	Virbac BVT
	*Escherichia coli* F5	Enteric disease	Antigen MICG	Virbac BVT
	*Escherichia coli* CS31A	Enteric disease	Antigen MICG	Virbac BVT
	Orthopoxvirus	Orthopox (the family of Smallpox)	Antigen MICG	Virbac BVT
	Rotavirus	Enteric disease	LFA (singleplex/multiplex)	Eurofins
	*Y. pestis*	Plague		
	*F. tularensis*	Tularemia	Antigen MICG	Virbac BVT
			LFA (singleplex/multiplex)	Eurofins
			LFA (singleplex/multiplex)	Eurofins

◇ This is not a comprehensive list of all commercially available POCT assays. Please refer to the individual manufacturer's websites. Some of the POCT assays are available in only certain countries.

# Companion and food animals.

MICG, Membrane immunochromatography.

**Table 3:** List of manufacturers' of POCT assays for infectious diseases in companion and food animals^*^.

**No**.	**Manufacturer**	**Website**
1	Alere (now Abbott)	https://www.globalpointofcare.abbott/en/products-solutions/product-catalog.html
2	Becton Dickinson	https://www.bd.com/en-us/products-and-solutions/products/product-families/bd-directigen-immunoassay-test-kits
3	Biogal	https://www.biogal.com/products/
4	BioNote	https://www.bionote.com/veterinary-products
5	Eurofins	https://www.eurofins-technologies.com/products/veterinary-diagnostics.html?p=2&product_list_mode=list&product_list_order=position
6	Fassissi GmbH	https://www.fassisi.de/produkte/
7	GeneReach	https://www.genereach.com/index.php
8	IDEXX	https://www.idexx.com/en/veterinary/products/snap-tests/
9	Immy	https://www.immy.com/
10	MEGACOR Diagnostik GmbH	https://www.megacor.at/veterinary.html
11	Midland BioProducts	https://www.biotech-careers.org/company/nittobo-america-midland-bioproducts
12	Quidel Corporation	https://www.quidel.com/search/node/influenza%20A%2BB
13	Tetracore	https://tetracore.com/product-category/domestic-preparedness/
14	Virbac BVT	https://bvt.virbac.com/en/home/diagnostic-solutions/pour-le-veterinaire-praticien.html
15	Zoetis	https://www.zoetis.com/products-and-science/diagnostics

* This is not a comprehensive list of manufacturers that offer POCT assays.

## Antibody based assays

There are several POCTs currently in use that detect antibodies against pathogens that cause animal diseases. These tests are used for disease surveillance as well as for diagnosis. A number of commercial kits are available for in clinic testing of antibodies against various disease pathogens in companion animals. One major disadvantage of antibody based POCT is the inability of these tests to differentiate between response to vaccination and natural exposure. However, there are a limited number of assays that are available that can differentiate between antibodies from vaccination and natural infections such as the FIV antibody POCT, “Witness” manufactured by Zoetis or “AntigenRapid” manufactured by BioNote (19).

Bergmann et al. (20) compared four commercially available POCTs to detect canine parvovirus (CPV) antibodies in dogs. Using virus neutralization as the reference standard test, they compared ImmunoComb Canine VacciCheck ELISA (Biogal Galed Labs), TiterCHEK CDV/CPV ELISA (Zoetis), ASTest CDV-CPV Ab Lateral Flow Immunoassay (MEGACOR Diagnostik GmbH), and CanTiCheck Lateral Flow Immunoassay (Fassisi GmbH). The data demonstrated that all the four POCTs were reliable in their performance, but FASTest had the highest sensitivity and CanTiCheck has the highest specificity (20). A similar study was conducted to compare POCTs by the same commercial vendors to detect canine distemper virus antibodies in dogs (21). The four POCTs examined had low specificity and variable sensitivity to detect CDV antibodies in sick dogs (21). An ImmunoComb POCT is available for the detection of antibodies against CPV, CDV, and canine adenovirus (CAV). A study to compare this POCT with virus neutralization assay for their performance in detecting CAV antibodies showed that though the POCT was a convenient platform, it had lower specificity for the detection of both CAV-1 and CAV-2 antibodies (22).

A POCT LFA, sona Coccidioides (IMMY, Norman, OK) was considered as a reliable POCT for the detection of antibodies against *coccidioides* in dogs (12). The positive percentage agreement with the current diagnostic standard, immunodiffusion assay was 88.9% and the negative percentage agreement was 100% (12).

In cattle, a milk test for antibodies to *Brucella* was developed and evaluated by Liebes et al. (23) using chemiluminescence immunosensors. They used silane-benzophenone as a coupling agent with killed *Brucella* in suspension to detect and quantify anti-Brucella IgG antibodies. The assay performance was superior to conventional ELISA with a limit of detection at 0.207 μg/ml (23). Montrose et al. (24) demonstrated the utility of a chip nanowire based immunosensor as a POCT by developing an electrochemical immunosensor device for the detection of bovine viral diarrhea virus (BVDV) antibodies in serum. The assay was able to detect the antibodies at 10 μg/ml level in 20 min. Biosensor based POCTs have also been developed for the detection of other pathogens including avian influenza virus, fowl adenovirus, *Brucella anthracis, Brucella melitensis, Salmonella*, and *Streptococcus suis* (25–34). Pen side tests and devices are available for testing the quality of colostrum by measuring immunoglobulins in the colostrum, such as Colostrometer, Brix refractometer, and an immunoassay kit by Midland BioProducts (Boone, IA) (35). Testing to facilitate breeding is an area where POCTs are often employed by producers or animal owners. An example is measuring of progesterone levels in milk or plasma or serum in cattle and horses. The test results help to make decisions on artificial insemination and embryo transfer. In equine practice, POCTs are used to detect immunoglobulin levels in foals (15, 36). Foals are prone to infectious diseases if they do not receive immunoglobulins through colostrum immediately after birth, and the immunoglobulin test helps to monitor the health of newborns (15).

Multi-analyte tests that can detect more than one antigen or antibody are also available on the market such as the SNAP tests by IDEXX Laboratories Inc. (Westbrook, ME) including SNAP 4Dx Plus (*Anaplasma phagocytophilum*/*A. platys* antibody, *Ehrlichia canis/E. ewingii* antibody, Lyme disease antibody, and heartworm antigen) (37), SNAP Feline Triple (FIV antibody, FeLV antigen, and feline heartworm antigen), and SNAP FIV/FeLV Combo (FIV antibody and FeLV antigen) (10, 37).

## Nucleic acid based assays

Nucleic acid amplification tests (NAATs) have been used as POCTs in both small animal and large animal diagnostics. Commercially available tests are offered by various manufacturers such as Fluxergy LLC (Irvine, CA), Cepheid (Sunnyvale, CA), and Sedia Biosciences (Portland, OR). Isothermal amplification is an ideal technique for a POCT (16, 17, 38–43). Isothermal implies that continuous amplification reactions occur at a constant temperature which is in contrast to conventional PCR which relies on thermal cycles (three temperature steps). The instrumentation for isothermal amplification is also simpler since the method only aims at reaching one constant temperature without the need for repeated thermal cycles. The whole process and test run times are significantly shorter compared to conventional PCR. While isothermal reactions may inherently lose some specificity in the hybridization events, this is compensated by optimizing conditions and through the addition of different enzymatic and biochemical reaction components—a modification which has proven diagnostically satisfactory across a broad range of applications (44).

Two examples of isothermal amplification assays are helicase-dependent amplification (HDA) and recombinase polymerase amplification (RPA). A recombinase polymerase amplification assay was developed and validated as a POCT to detect feline herpesvirus type 1 that used a lateral flow dip strip to read the results. The assay was comparable to regular PCR and was a reliable POCT for FHV-1 detection (45).

Loop mediated isothermal amplification (LAMP) is a commonly used platform in POCT (46). Gunther et al. (47) compared two reaction mixes, Isothermal Master mix from OptiGene Ltd. and PCRun Molecular Detection Mix from Biogal for their ability to detect feline coronavirus (FCoV) in an isothermal amplification platform using same set of primers. Data showed that both are reliable as POCTs in detecting FCoV from effusion samples with or without feline infectious peritonitis, but the sensitivity was less compared to RT-PCR (47).

A PCR based POCT was developed by Fluxergy for the detection of *Streptococcus equi* subspecies *equi*. Compared to RT-PCR, this POCT had a sensitivity of 89% and a specificity of 100% (36). Comparing POCTs for the diagnosis of equine abortions with standard laboratory RT-PCR assays showed that they were a reliable way of screening for reproductive pathogens in the equine industry. The data showed that *Chlamydia psittaci* real-time fluorometer LAMP had 100% sensitivity and 97.5% specificity when compared with RT-PCR while equine herpesvirus-1 (EHV-1) rtLAMP had 86.96% sensitivity and 100% specificity compared to EHV-1 RT-PCR (48). An insulated isothermal PCR (iiPCR) based POCT for equine herpesvirus-3, the causative agent of equine coital exanthema, had 98.82% agreement with RT-PCR when 85 perineal and genital swabs of mares and stallions were tested. The assay showed high sensitivity and specificity with a limit of detection comparable to RT-PCR (49). Isothermal nucleic acid amplification based POCTs are also available for African horse sickness, Western equine encephalitis, equine infectious anemia, equine influenza, equine viral arteritis, equine rhinopneumonitis (EHV-1), Hendra virus, Japanese encephalitis, Ross River virus, and West Nile virus as reviewed by Knox and Beddoe (50).

In bovine disease diagnostics, LAMP has been increasingly used as a POCT including the assays for detecting pathogens causing mastitis. POCTs are used in-line, in-parlor, and in the laboratory, both at the farm side and veterinary practice laboratory to diagnose mastitis in cattle (41, 51). Compared to PCR or rRT-PCR, LAMP assays are less affected by inhibitory substances in the milk. When coupled with LFAs, LAMP can be easily used at the farm by producers. However, there are some limitations in using LAMP for mastitis diagnosis including the heterogeneity of mastitis pathogens and resistance determinants, and the reduced capacity for multiplexing (41, 51). Yamazaki et al. (42) used a combination of EZ fast DNA extraction and LAMP to amplify bovine leukemia virus DNA from blood for the diagnosis of bovine leukemia. The assay was comparable to rRT-PCR in terms of sensitivity and specificity, and rapid with the extraction step taking <10 min to complete. Another LAMP based assay was developed and evaluated in cattle by Chen et al. (38) to detect capripox (CaPV) in skin lesions. They used trans-cleavage activity of CRISPR/Cpf 1 to recognize the target DNA to identify the amplification product of LAMP assay in the detection of CaPV.

An isothermal recombinase polymerase amplification (RPA) was developed and evaluated for the detection of infectious bovine rhinotracheitis virus (IBRV) from clinical samples (39). The assay used lateral flow dips trip (LFD) method to read the results. The RPA primers and probe targeted the UL52 region of IBRV and the turnaround time for the assay was 25 min. The assay results were comparable to SYBR Green-1 based PCR when validated using clinical samples such as feces, blood, nasal swabs, and tissues (39). Liu et al. (40) developed a multiple cross displacement amplification (MCDA) to detect *Mycobacterium tuberculosis* (MTB) in cattle. In this assay, hydroxy naphthol blue was used for the colorimetric detection of the amplification products. The assay showed high specificity and sensitivity and could be used as a POCT for MTB in bovine samples (40). POCTs are also available for clinical mastitis testing, for culture and microbial susceptibility testing (41, 51, 52).

## Tests in wild and exotic animals

There is a market need for developing and validating POCT for detecting pathogens from wild and exotic animals for early diagnosis of diseases. Around 75% of the emerging infectious diseases are zoonotic in nature (53), and more than 70% of those zoonoses originate from wild birds or animals (54). However, there are many challenges in developing and validating POCTs for wild and exotic animals. These challenges include lack of information on wildlife physiology and pathogen behavior, regulatory and logistic considerations about the collection of specimens from wildlife, incomplete information on sampled animals or species, and sampling challenges (55). A quantitative RT-PCR assay was developed by Tomaszewicz Brown et al. (56) for detecting canine distemper virus (CDV) from wildlife using a handled platform called Biomeme that performs qPCR with lyophilized shelf-stable reagents. The platform had comparable specificity with laboratory-based methods but had decreased diagnostic sensitivity (56). A point-of-care tuberculosis diagnostic kit for the detection of antibodies from wild animals was developed by Veerasami et al. (57) using pathogenic mycobacteria specific recombinant antigens and purified protein derivatives of pathogenic and non-pathogenic *Mycobacteria*. The kit was used to determine the seroprevalence of TB in wild animals including sloth bear, elephants, wild bear and wild dogs (57). A LAMP assay was developed and validated for the detection of *Chlamydia* from wild koalas by Hulse et al. (58) as a POCT. The assay showed 100% specificity with qPCR results and had a limit of detection (LOD) of 44 IFU/ml.

## Challenges in quality assurance and quality control

There is a rapid influx of POCT devices and methods on the market with technological advancements (55, 59, 60). Often, POCTs are not held to the same standards as of regular laboratory diagnostic tests or assays. There is no adequate validation standards or regulatory requirements. As a result, there is no proper approval procedures to ensure quality of results (55, 61). The World Organization for Animal Health (WOAH) provides an official validation and certification of animal diagnostic tests including POCT (62). The WOAH also has a register to keep track of veterinary diagnostic kits. However, this is not mandatory in most countries and not regulated by WOAH. Most of the small-scale POCT manufacturers do not consider registering their products because of cost and marketability concerns (55).

While examining the current limitations of the POCT validation and regulatory processes, Hobbs et al. (55) discussed lack of consistency and transparency of POCT validation data, lack and limitations of filed validation studies, and difficulties in conducting full validation of POCTs for certain pathogens as main contributing factors.

Though there are several concerns about quality control in veterinary POCTs, not much guidance is available for reference. In this context, the American Society for Veterinary Clinical Pathology (ASVCP) developed quality assurance guidance for veterinary POCT, which was published in 2013 (59). Their main recommendations were to take a formalized approach to POCT, use written policies, standard operating procedures, forms and logs, to train the operator including periodic assessment of skills, ask for the analytical performance of instruments, use properly established or validated reference intervals and ensure accurate reporting of results (59). These guidelines were not meant to be all inclusive, but mainly for veterinarians or technicians to improve POCT standards in their research or clinical facilities.

## Advantages and disadvantages of POCT

There are several advantages to POCT. As the name indicates, tests can be performed at the pen-side without the need for going to an animal clinic or laboratory, so it's a convenient way of testing by animal owners or caretakers. This is especially beneficial for rural farmers with limited access to veterinary services. Faster turnaround time is another advantage that helps to make evidence-based decisions on treatment and management of diseases. Early detection helps to mitigate and minimize negative outcomes. Most of the POCTs are easy to use and require minimum training of the end users. For food animal producers, having POCTs help them manage heard health better and help prevent the spread of diseases.

The main disadvantage in most of the cases is poor quality control for POCTs that includes insufficient validation, lack of controls, lack of proficiency testing and low-throughput designs of the POCTs. This applies to diagnostic test kits, reagents, devices and the actual performance of testing. Affordability and cost-effectiveness are other factors, which are even more significant in food animal disease diagnosis than in companion animal disease diagnosis. Simplicity of the assay and portability of devices are important for POCTs to be attractive, and the lack of these features would be disadvantageous for widespread acceptance of the platform. In resource limited communities and remote areas, costly and equipment-dependent POCTs would keep customers away from using the assays. Availability of thermostable reagents would be another factor that would drive the market favorably for POCTs. For POCTs to be successful, the end users should be able to use the assays and devices with minimal training. Extensive preparatory work and complicated and long test procedures would make POCTs less attractive to the end users.

When it comes to analyte being tested or technology being used, each assay has its own advantages and disadvantages. In general, antigen and antibody-based assays are easy to develop, user-friendly, and simple to perform, but their sensitivity and specificity are low compared to nucleic acid-based assays. A number of LFAs are used as POCTs. They are simple to use, easy to read, cheap, and rapid. They don't need refrigeration and have a longer shelf-life They are ideal in resource-limited countries. However, LFAs often require a secondary assay to confirm results, and so they mainly serve as screening tests (63). Nucleic acid-based POCTs have high sensitivity and specificity. They also have rapid turnaround time. The main disadvantages of NAT as POCTs include high cost for development of assays, sample preparation or extraction of nucleic acid in certain cases, and the need for training and instrumentation for performing the assays (64, 65).

Lack of sufficient investments in POCT development is also a problem since companies may not be willing to take a risk if there is no optimal return on investment. Conducting a full-scale filed study to take all variables into account for test validation is also a challenge. However, there is more awareness among animal owners regarding the availability and advantages of POCTs and as research and development in the POCT field is progressing, products with more advantages than disadvantages are coming out on the market in a steady pace.

## Concluding remarks and future directions

The ongoing COVID-19 pandemic augmented the rapid development of a plethora of POCT technologies and methodologies. Miniaturized isothermal amplification tests, lateral flow assays, chemiluminescence, nanoparticle-based assays became available on the market (66). There was a sea change in the use of POCTs in the human disease diagnostics post-COVID-19 with advanced technologies coming to the market including high-performing biosensors and chips, CRISPR/Cas technology and paper fluidic devices. A paper fluidic device-based assay, FnCas9 editor-linked uniform detection assay (FELUDA), was developed recently in India to detect SARS-CoV-2 (67). This trend of using advanced technologies is getting reflected on the animal disease diagnostic side as well. There is a demand for miniaturized NAAT based devices on veterinary diagnostics market that are automated, high throughput and affordable. Smartphone based POCTs are also rapidly emerging on the market supported by increasing research activities in this area in recent years (68). With evolving technology, high accuracy and sensitivity are shown by platforms that combine biosensors, smartphones, and other accessories. Such platforms are affordable and easy to use in resource limited areas (68).

Though NAAT based assays using miniaturized PCR devices is gaining popularity, there are several limitations to this platform including challenges in quantitating the analytes, nucleic acid extraction or purification and concentration of analytes, and thermocycling for PCR amplification. Isothermal amplification is a robust and simple platform well-suited for POCT. It does not need sophisticated instrument and the amplified product can be read using colorimetry, fluorescence or LFA. Several assays to detect infections agents are underway using this technique. The main diagnostic methods that use isothermal amplification include LAMP, helicase-dependent amplification (HAD), recombinase polymerase assay (RPA), cross-priming amplification (CPA), strand exchange amplification (SEA), rolling circle amplification (RCA), Multiple displacement amplification (MDA), and nucleic acid sequence-based amplification (NASBA) (43, 69).

Lateral flow assays and devices have also gone through several transitions and upgrades including addition of colloidal gold particles to improve specificity and to make them more advanced and efficient (9). Their major advantages include scalability, low cost, easy to use, and quick turnaround for development and approval (9).

Multiplex POCTs that could help diagnose animal diseases with a syndromic or system-based approach with a single test would be a *win-win* for the end users and veterinarians/clinicians who treat diseases or make clinical management decisions (70). The multiplex POCTs will serve the animal disease diagnostic market well in the future as a rapid, cost-effective approach that uses less volume of samples and helps make therapeutic or control decisions. Such an assay coupled with high throughput capability would be a helpful tool for disease surveillance in food and fiber animals and beneficial in disease diagnostics to rapidly respond to disease outbreaks. Multiplex assays have the potential to advance precision agriculture and precision medicine through cutting-edge diagnostics that can be done at the pen-side or bed-side. More work needs to be done in optimizing multiplex platforms and devices to ensure high sensitivity and specificity. Multiplexing coupled with artificial intelligence and deep learning or machine learning (71) algorithms could pave the way for the creation of more powerful and sophisticated POCT platforms that can be used in animal and human disease diagnostics.

## Author contributions

All authors listed have made a substantial, direct, and intellectual contribution to the work and approved it for publication.

## Conflict of interest

The authors declare that the research was conducted in the absence of any commercial or financial relationships that could be construed as a potential conflict of interest.

## Publisher's note

All claims expressed in this article are solely those of the authors and do not necessarily represent those of their affiliated organizations, or those of the publisher, the editors and the reviewers. Any product that may be evaluated in this article, or claim that may be made by its manufacturer, is not guaranteed or endorsed by the publisher.
